# Editorial: NAR Awards 2015

**DOI:** 10.1093/nar/gkv1360

**Published:** 2015-12-10

For the tenth year running, NAR and Oxford Journals have provided sponsorship and awarded travel bursaries and prizes to students and junior scientists in recognition of their outstanding achievements. This year, sponsorship and prizes were given at 13 different meetings. A list of the awards is presented below. Our warm congratulations go to the prize winners.

**Student Travel Bursary**

**2015 Mammalian DNA Repair Gordon Research Conference**

Ventura, CA, USA

February 8–13, 2015

**Poster Prizes**

**DNA Repair in Chromatin Symposium**

Pullman, WA, USA

May 21–23, 2015

**Amelia J. Hodges** (School of Molecular Biosciences and Center for Reproductive Biology, Washington State University, USA)

**John W. Smerdon** (Department of Physiology and Cellular Biophysics, Columbia University, USA)

**International Meeting on Quadruplex DNA**

Bordeaux, France

May 26–28, 2015

**Louis K. M. Chan** (Cambridge University, UK)

Small Molecule Destabilisation of Intermolecular G-Quadruplexes

**Daniela Verga** (University of Konstanz, Germany)

G-quadruplex DNAzyme-modified Nucleotides for Diagnostic Applications

**RNA Stability 2015 Conference**

Estes Park, CO, USA

June 1–4, 2015

**Eva Kowalinski** (Max Planck Institute of Biochemistry, Germany)

**11^th^ Nucleic Acids Research Forum**

London, UK

July 10, 2015

**Jonathan Burns** (National Physics Laboratory / UCL, UK)

**International Conference on Intelligent Systems for Molecular Biology/ European Conference on Computational Biology**

Dublin, Ireland

July 10–14, 2014

**Robin van der Lee** (Radbound University Medical Center, Netherlands)

**Jonas Ibn-Salem** (Johannes Gutenberg University Mainz, Germany)

**7th New England Biolabs Meeting on DNA Restriction and Modification**

Gdansk, Poland

August 24–29, 2015

**Jasmina Dikic** (University of Leipzig, Germany)

**Ewa Wons** (University of Gdansk, Poland)

**42nd International Symposium on Nucleic Acids Chemistry (ISNAC 2015)**

Hyogo, Japan

September 23–29, 2015

**Ken Yamada** (Tohoku University, Japan and McGill University, Canada)

Effects of Sugar Conformational Preferences during Nucleotide Excision by HIV-1 Reverse Transcriptase

**Masayuki Tera** (Suntory Foundation for Life Science, Japan)

Development of Barbering Dimmer as a Turn-on Fluorescent G-quadruplex Ligand

**Environmental Mutagenesis and Genomics Society Meeting**

New Orleans, LA, USA

September 26–30, 2015

**Student New Investigator Poster Session support**

**Genome Engineering (European Lab Automation)**

Hanover, Germany

October 7–8, 2015

**Jamal Alzubi** (Institute for Cell and Gene Therapy, Freiburg, Germany)

**Annual Meeting of the Oligonucleotide Therapeutics Society**

Leiden, the Netherlands

October 11–14, 2015

**Umar Burki** (Newcastle University, UK)

In vivo Pharmacokinetic and Biodistribution Profile of Peptide-Conjugated Phosphorodiamidate Morpholino Oligonucleotides

**Alexandre Debacker** (University of Southampton, UK)

Optimized DNA-PNA Chimeras for Silencing Structured RNAs

**Anna Dysko** (University of Oxford, UK)

Synthesis of Modified Oligonucleotides Containing Triazole Backbones

**Ellen Gyssels** (University of Ghent, Belgium)

Covalently Locking Nucleic Acid Structures by the Inducible Furan Cross-link Strategy: New Applications

**Marcel Hollenstein** (University of Bern, Switzerland)

General Compatibility of Nucleoside Triphosphates with Rolling Circleamplification

**Satish Jadhav** (University of Turku, Finland)

Synthesis of Carbohydrates/Bisphosphonate-Oligonucleotide Conjugates and their PET Imaging

**Silvana Jirka** (Leiden University Medical Center, Netherlands)

Evaluation of 2′- deoxy-2′-fluoro Antisense Oligonucleotides for Exon Skipping in Duchenne Muscular Dystrophy

**Yifat Oren** (The Alexander Silberman Institute of Life Sciences, Israel)

Restoration of cftr Function by Antisense Oligonucleotide Splicing Modulation

**Maire Osborn** (RNA Therapeutics Institute, UMass Medical School, USA)

Polyunsaturated Fatty Acid-siRNA Conjugate Targeting Huntingtin (Htt) mRNA Shows Prolonged Efficacy, Minimal Toxicity, and Broad Distribution in Mouse Brain

**Dina Polyak** (Sackler School of Medicine Tel Aviv University, Israel)

Hindering Tumor Growth by Polymer-based Targeted Delivery of Oligonucleotides

**Jurriën Prins** (Leiden University Medical Center, Netherlands)

Post-Transcriptional Guidance of Monocyte to Macrophage Differentiation by the RNA-Binding Protein Quaking

**Lodewijk Toonen** (Leiden University Medical Center, Netherlands)

Ataxin-3 Exon Skipping as a Treatment Strategy for Spinocerebellar Ataxia Type 3

**Tirsa Van Westering** (University of Oxford, UK)

Cmah-/-mdx Mouse Early Onset Cardiac Phenotype and Correction of Dystrophin by Peptide-PMO for Duchenne Muscular Dystrophy

**Ann Fiegen Durbin** (Harvard University and MIT, USA)

Signal Interrupted: How RNAs Containing Modified Nucleotides Suppress RIG-I Activation

**6th Argentine Conference on Computational Biology and Bioinformatics (CAB2C)**

Bahía Blanca, Argentina

October 14–16, 2015

**Diego Javier Zea** and Cristina Marino Buslje (Fundación Instituto Leloir, Argentina)

**Estefanía Mancini**, Sabrina Sanchez, Rubén Gustavo Schlaen, Andrés Romanowski, Esteban Hernando and Marcelo Yanovsky (Fundación Instituto Leloir, Argentina)

**Alexander Monzon**, Diego Zea, María Silvina Fornasari, Silvio Tosatto and Gustavo Parisi (Universidad Nacional de Quilmes, Argentina)

**10^th^ International Symposium on Aminoacyl-tRNA Synthetases**

Barcelona, Spain

October 18–22, 2015

**Ita Gruic-Sovulj** (University of Zagreb, Hungary)

**Renaud Geslain** (College of Charleston, USA)

